# Association between osteoprotegerin gene T950C polymorphism and osteoporosis risk in the Chinese population: Evidence via meta-analysis

**DOI:** 10.1371/journal.pone.0189825

**Published:** 2017-12-18

**Authors:** Shouyi Li, Huiqiang Jiang, Ningke Du

**Affiliations:** 1 Department of rehabilitation, People's Hospital of Jilin Province, Changchun, China; 2 Department of Nursing, Children's hospital of Changchun, Changchun, China; State University of New York, UNITED STATES

## Abstract

Osteoporosis has been reported to be at least partially developed in response to functional polymorphisms of the osteoprotegerin (OPG). However, conflicting results have been found. This meta-analysis aimed to provide an assessment of the relationship between the risk for developing osteoporosis and OPG T950C polymorphism in the Chinese population. Studies to be analyzed were identified with the literature search in PubMed, Embase, Web of Science, the Cochrane Library, and the Chinese National Knowledge Infrastructure during May 2017. Seven case-control studies that included a total of 1850 osteoporosis cases and 3074 controls were assessed in this meta-analysis. Overall, no significant associations could be detected between OPG T950C polymorphism and osteoporosis when all included studies were pooled into this meta-analysis. In a subgroup analyses, OPG T950C polymorphism was significantly associated with the osteoporosis risk in South China (CC+TC vs. TT: OR = 1.34, 95% CI = 1.17–1.54; CC vs. TC+TT: OR = 0.79, 95% CI = 0.69–0.95) and for studies that included postmenopausal osteoporosis (CC vs. TC+TT: OR = 0.78, 95% CI = 0.64–0.94) or hospital-based controls (CC vs. TC+TT: OR = 0.81, 95% CI = 0.68–0.96). In conclusion, the results of this meta-analysis suggest that OPG T950C polymorphism might be associated with an increased osteoporosis risk in the Chinese population.

## Introduction

It has previously been estimated that 30% of women and 12% of men were affected by osteoporosis [1]. Previous studies have suggested osteoporosis to be a multifactorial genetic disease in which genetic determinants are typically modulated by hormonal, environmental, and nutritional factors [2]. To date, several candidate genes, such as osteoprotegerin (OPG), have been reported to be associated with both bone mineral density (BMD) and osteoporosis [3–6]. Among these candidate gene polymorphisms, OPG T950C is one of the most widely studied genetic variant for osteoporosis. Langdahl et al. first investigated the link between OPG T950C polymorphism and osteoporosis in a Caucasian population in 2002 [7]. Furthermore, a large number of epidemiological studies have been conducted to assess the relationship between OPG T950C polymorphisms and the individual susceptibility to osteoporosis. However, the obtained results have been inconsistent. These different results might have been caused by racial and regional differences in the studied patients, as well as by the limitation of the number of patients per study. To reduce the influence of diverse genetic backgrounds, here, we performed a meta-analysis based on the Chinese population only to assess the relationship between OPG T950C polymorphism and the risk for developing osteoporosis.

## Materials and methods

### Identification of eligible studies

We searched for studies that examined the relationship between the OPG T950C polymorphism and osteoporosis, using the following databases: PubMed, Embase, Web of Science, the Cochrane Library, and the Chinese National Knowledge Infrastructure. The utilized keywords were (OPG or osteoprotegerin or TNFRSF11B) and osteoporosis and polymorphism and (Chinese or China) and the search was last updated in May 2017. Additional records were identified via manual search and the utilized inclusion criteria were: (1) studies that described the association between OPG T950C polymorphism and the development of osteoporosis, (2) studies that used sufficient genotypic data to calculate the odds ratio (OR), (3) all participants were of Chinese population. The following exclusion criteria were defined: (1) studies that contained incomplete data, (2) case reports, (3) editorial articles, (4) review articles, and (5) meeting abstracts.

### Data extraction

Information was carefully extracted from all eligible publications independently by two authors (Li S and Jiang H) according to the inclusion criteria. The titles and abstracts of all potentially relevant articles were screened firstly. Full articles were then scrutinized if the title and abstract were ambiguous. Disagreements were resolved through a discussion between the two authors. The following characteristics were collected for each study: name of first author, year of publication, source of controls, geographic area, sample size, and available genotypic data for OPG T950C.

### Statistical analysis

The relationship between OPG T950C polymorphism and the development of osteoporosis was evaluated via calculating pooled ORs, based on individual ORs. A meta-analysis examined the overall relationship of the C allele with the risk for osteoporosis development relative to the T allele; and the contrast of homozygotes CC versus TT, the contrast CC versus (TC+TT), and the contrast (CC+TC) versus TT. The heterogeneity between the utilized studies and Hardy-Weinberg equilibrium (HWE) expectations in controls were tested with a Chi-square-based Q-test [8]. Either the fixed-effect model (by Mantel-Haenszel) or the random-effect model (by DerSimonian and Laird) were selected to summarize combined ORs and their 95%Cis according to the previously obtained results of the heterogeneity test. A fixed effects model was used when there was no heterogeneity among these studies; otherwise, the random-effects model was used. Subgroup analyses were conducted by integrating control sources, geographic area, and subject type. Sensitivity analyses were conducted via comparison of the results of fixed-effects model and random-effects model. All statistical analyses were conducted with Stata, version 12 (StataCorp LP, College Station, TX). P-values below 0.05 were considered to indicate statistically significant differences.

## Results

### Description of included studies

**Fig 1:**
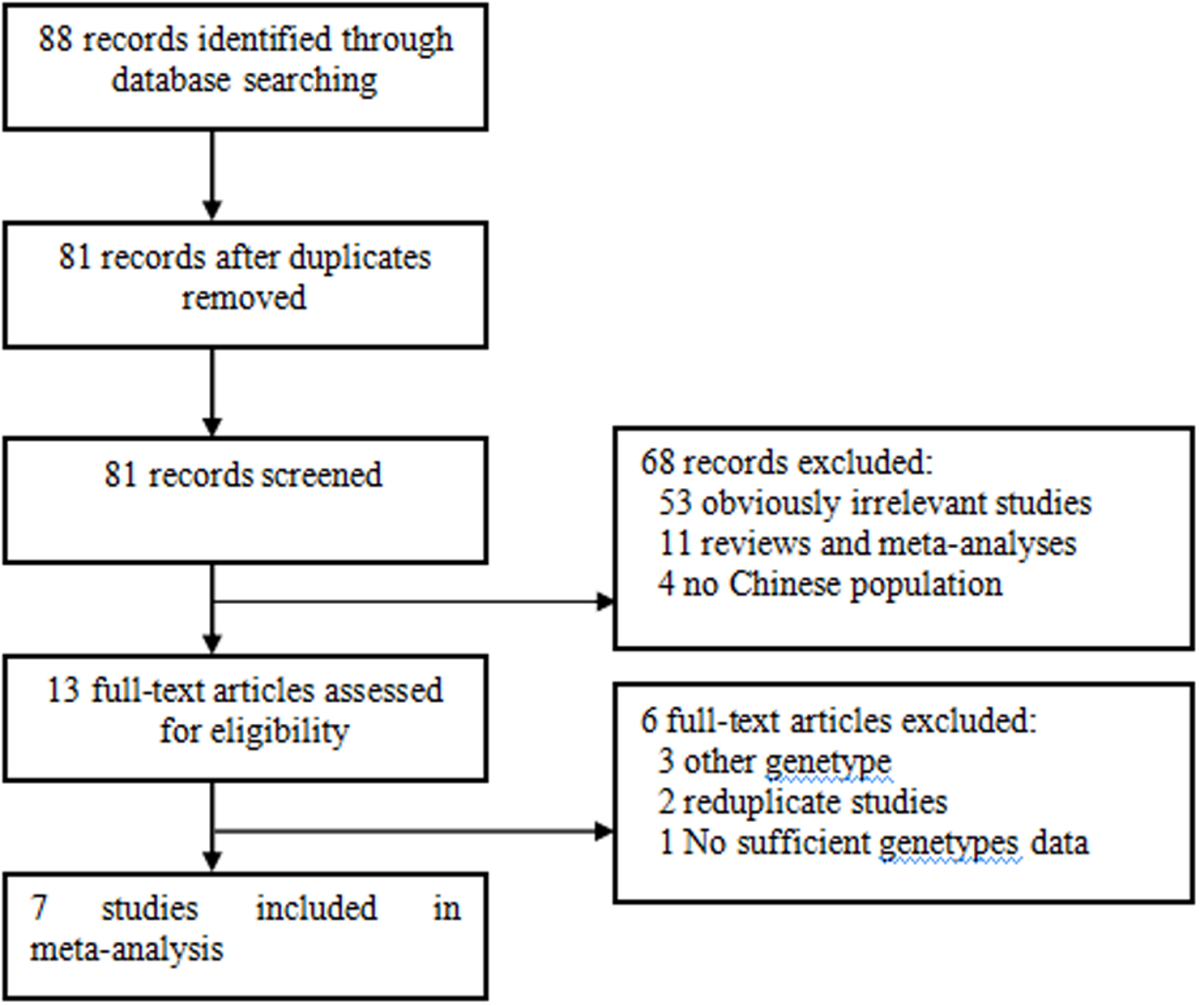
Literature search flow diagram.

Eighty-eight relevant publications were identified with the outlined search strategy. In agreement with the outlined inclusion criteria, seven case-control studies [9–15] met our inclusion criteria; however, 81 studies had to be excluded from the meta-analysis. A flow chart of the process of study selection and specific reasons for exclusions are shown in Fig 1. A total of 4924 subjects were included in this meta-analysis, including 1850 cases of osteoporosis and 3074 control cases. The publication years of the assessed studies ranged from 2005 to 2012. The source of the controls of one of the included studies was based on the population. S1 Table lists the characteristics of the included studies.

### Results of meta-analysis

**Fig 2:**
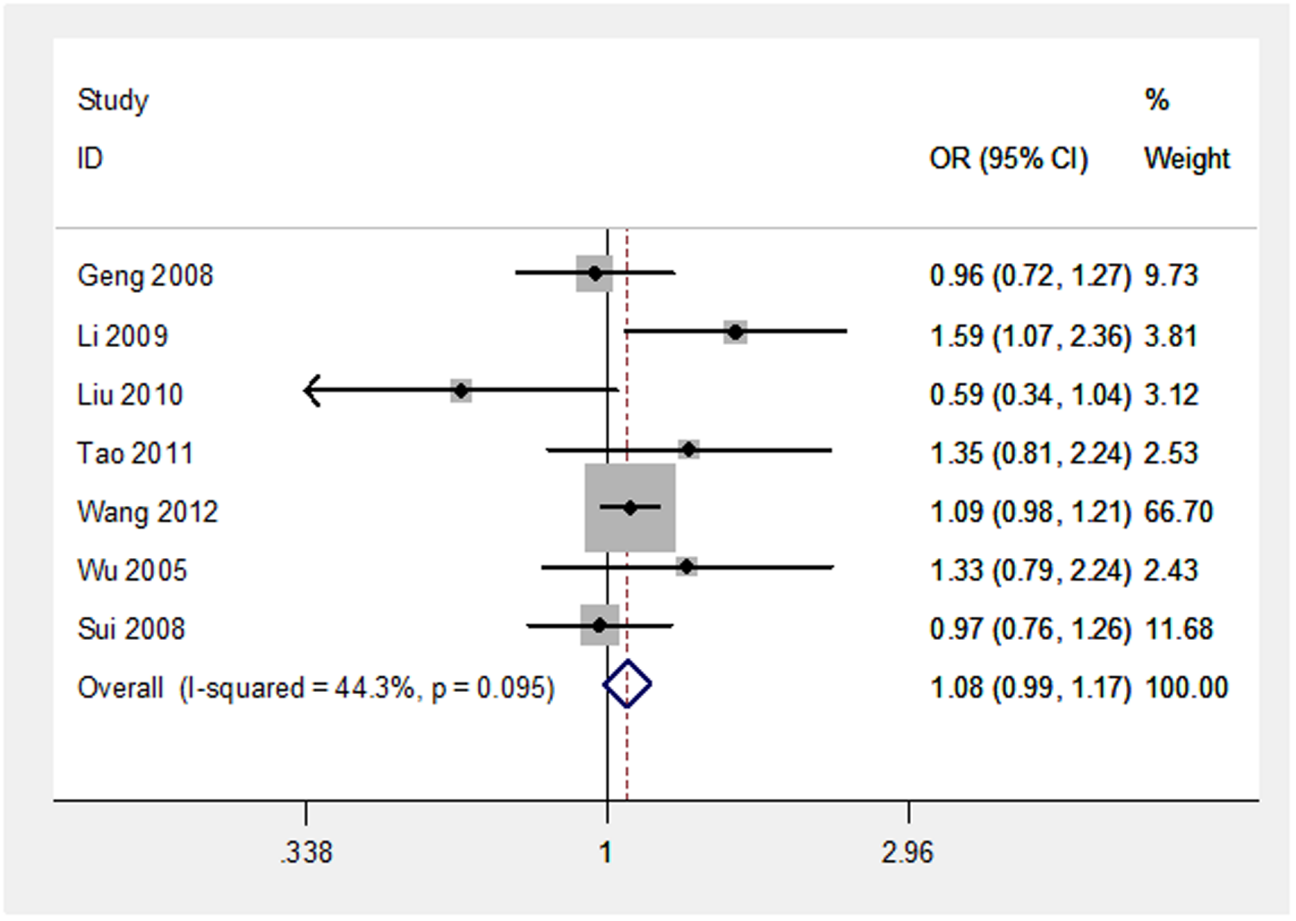
Forest plot for the association between OPG T950C polymorphism and the risk to develop osteoporosis under the allele fixed-effect model.

An evaluation of the links between OPG T950C polymorphism and the risk of osteoporosis development are summarized in Table 1. Throughout the overall analyses, no significantly elevated risk of osteoporosis development could be detected in any of the analysis models (Table 1, Fig 2). The stratified analysis (by both subject type and control sources) indicated a significant decreased association for postmenopausal women and studies with hospital-based controls (CC vs. TC+TT: OR = 0.78, 95% CI = 0.64–0.94; CC vs. TC+TT: OR = 0.81, 95% CI = 0.68–0.96). This suggests that the OPG T950C CC genotype may be a protective factor for postmenopausal osteoporosis. The stratified analysis via geographic area showed a significantly increased osteoporosis risk was found for the population of South China via the dominant model (CC+TC vs. TT: OR = 1.34, 95% CI = 1.17–1.54), while a decreased risk was found in South China via the recessive model (CC vs. TC+TT: OR = 0.79, 95% CI = 0.69–0.95).

**Table 1:** Association of the OPG T950C polymorphism on the propensity to develop osteoporosis.

Analysis model		n	ORr(95%CI)	ORf(95%CI)	P_h_
C vs. T	Total analysis	7	1.08 (0.92–1.26)	1.08 (0.99–1.17)	0.095
	Hospital-based	6	1.03 (0.90–1.19)	1.06 (0.97–1.15)	0.228
	South China	4	1.09 (0.99–1.19)	1.09 (0.99–1.19)	0.561
	North China	3	1.00 (0.63–1.60)	1.04 (0.85–1.27)	0.014
	Postmenopausal women	5	1.04 (0.87–1.25)	1.07 (0.97–1.17)	0.168
CC vs. TT	Total analysis	7	1.08 (0.77–1.51)	0.99 (0.82–1.19)	0.087
	Hospital-based	6	0.94 (0.74–1.20)	0.94 (0.77–1.14)	0.333
	South China	4	0.96 (0.78–1.19)	0.97 (0.78–1.19)	0.412
	North China	3	1.00 (0.41–2.45)	1.08 (0.73–1.59)	0.019
	Postmenopausal women	5	0.96 (0.66–1.38)	0.93 (0.76–1.15)	0.220
CC vs. TC+TT	Total analysis	7	1.08 (0.75–1.56)	0.87 (0.73–1.02)	0.011
	Hospital-based	6	0.89 (0.68–1.16)	0.81 (0.68–0.96)	0.197
	South China	4	0.98 (0.68–1.53)	0.79 (0.69–0.95)	0.098
	North China	3	1.16 (0.59–2.29)	1.15 (0.83–1.60)	0.038
	Postmenopausal women	5	0.90 (0.62–1.29)	0.78 (0.64–0.94)	0.165
CC+TC vs. TT	Total analysis	7	1.11 (0.85–1.47)	1.27 (1.12–1.44)	0.026
	Hospital-based	6	1.07 (0.78–1.46)	1.27 (1.11–1.44)	0.015
	South China	4	1.24 (0.93–1.66)	1.34 (1.17–1.54)	0.133
	North China	3	0.90 (0.50–1.59)	0.96 (0.70–1.31)	0.075
	Postmenopausal women	5	1.08 (0.74–1.59)	1.30 (1.14–1.49)	0.015

ORr: Odd ratio for random-effects model; ORf: Odd ratio for fixed-effects model; P_h_: *P* value for heterogeneity test; South China including Chongqing, Guangdong, Chongqing and Shanghai; North China including Heilongjiang, Beijing, and Shandong.

### Sensitive analysis

In order to compare the difference and evaluate the sensitivity of the meta-analyses, we used both models (the fixed-effects model and random-effects model) to evaluate the stability of the meta-analysis. The pooled results were compared between fixed-effects and random-effects models, thus evaluating the sensitivity of the meta-analysis. All significantly pooled ORs were substantially altered. Consequently, results of the sensitivity analysis suggest that the data of this meta-analysis were rather unstable (Table 1).

## Discussion

Osteoporosis is a relatively common skeletal disorder, which is characterized by low bone mass and an accompanied deterioration of the bone microarchitecture with an increased propensity to fracture [16]. Recently, a study suggested that the receptor activator of the nuclear factor kappa B (RANK), the RANK ligand, and OPG regulate both osteoclast formation and activity [17]. The OPG gene (which encodes OPG) seems to be an attractive candidate gene for the development of osteoporosis. Polymorphism of the OPG T950C gene is one of the chief subtypes of OPG gene polymorphisms. To date, numerous studies have been conducted to identify whether a polymorphism in OPG T950C provided a genetic determinant for the development of osteoporosis. Unfortunately, conflicting results have been obtained in the conducted studies. Therefore, we conducted this updated meta-analysis to estimate the relationship between OPG T950C polymorphism and the inclination to develop osteoporosis, only using the Chinese population to decrease the impact of the genetic background. A total of seven studies with 1850 cases of osteoporosis and 3074 control cases were evaluated for this meta-analysis. The results show that polymorphism of OPG T950C was significantly linked to the risk for the development of osteoporosis in South China and in studies that investigated postmenopausal osteoporosis or hospital-based controls.

During the past decade, numerous published meta-analyses focused on polymorphisms of OPG T950C and a potential link to the risk for the development of osteoporosis [18–20]. Lee et al. [18] performed a meta-analysis that included eight studies and the authors reported the G1181C polymorphism to be associated with lumbar BMD in both Europeans and Asians, as well as with femoral neck and total hip BMD (in Europeans only); however, the authors reported no associations between A163G and T950C polymorphisms and BMD. In 2014, Guo et al. [19] conducted a comprehensive evaluation of the link between seven widespread OPG genetic polymorphisms (T149C, A163G, G209A, T245G, T950C, G1181C, and C1217T) and the risk for the development of osteoporosis. Their results indicated that OPG A163G and G1181C were significantly associated with the risk for the development of osteoporosis. A further meta-analysis included four studies and suggested that a polymorphism of OPG T950C decreased the risk for the development of osteoporosis [20]. Including more studies that have been conducted with a Chinese population and subgroups will strengthen the presented meta-analysis. To some extent, the obtained results matched those reported in previous studies. Therefore, our obtained results indicated that OPG T950C polymorphism might be associated with a risk of osteoporosis development in the Chinese population.

Our meta-analysis has several strengths: First, we strictly followed predefined inclusion and exclusion criteria to reduce selection bias as much as possible. Second, inclusion of non-English language reports was important to minimize a major potential threat for the validity of meta-analysis-publication bias and risks related to language bias. Third, we investigated the influence of the geographic area with regard to the risk to develop osteoporosis in relation to OPG T950C polymorphism.

The associated limitations should also be pointed out: First, the conducted (ethnic-specific) meta-analysis included data from a single ethnic group only; therefore, our results are only applicable to this particular ethnic group. Second, the obtained results of the sensitivity analysis were relatively unstable, which may potentially influence our obtained conclusion. Third, the etiology of osteoporosis is relatively complex and is mediated by activities of numerous genes. A single gene or a single nucleotide polymorphism might impact the osteoporosis risk less than what has been suggested so far. Finally, due to limitations of the funnel plotting (which requires a range of studies), we could not evaluate publication bias for this meta-analysis.

In conclusion, this meta-analysis indicated that OPG T950C polymorphism might be linked to the risk for developing osteoporosis in the Chinese population. However, due to the limitations of this study, the presented results and conclusions should be interpreted cautiously and further large-scale studies are warranted to confirm the findings.

## Supporting information

S1 Table Characteristics of the studies that were included in the meta-analysis. (DOCX)

S2 Table Meta-analysis on genetic association studies checklist. (DOCX)
