# Supplementary material for: Association between osteoprotegerin gene T950C polymorphism and osteoporosis risk in the Chinese population: Evidence via meta-analysis
Source: PLoS One. 2017 Dec 18;12(12):e0189825. doi: 10.1371/journal.pone.0189825 (PMC5734735; doi:10.1371/journal.pone.0189825)
Supplement: S1 Table — (DOCX) [file pone.0189825.s001.docx]

| References | Control source | Geographic area | Subject type | Genotyping method | Case | | Control | | Cases | | |  | Controls | | |  | HWE | |
| --- | --- | --- | --- | --- | --- | --- | --- | --- | --- | --- | --- | --- | --- | --- | --- | --- | --- | --- |
|  |  |  |  |  | Number | Age | Number | Age | TT | TC | CC |  | TT | TC | CC |  | χ² | *P* |
| Geng 2008 | Hospital-  based | Chongqing | Postmenopausal women | PCR-RFLP | 186 | 41-68 | 214 | 41-64 | 70 | 76 | 40 |  | 72 | 100 | 42 |  | 0.47 | 0.495 |
| Li 2009 | Population  -based | Shandong | Men | PCR-RFLP | 98 | 70.9± 8.2 | 101 | 71.0± 7.5 | 25 | 39 | 34 |  | 33 | 49 | 19 |  | 0.01 | 0.914 |
| Liu 2010 | Hospital-  based | Beijing | Postmenopausal women | PCR-RFLP | 50 | 77.6± 8.0 | 50 | 77.5± 7.6 | 18 | 23 | 9 |  | 9 | 28 | 13 |  | 0.81 | 0.368 |
| Tao 2011 | Hospital-  based | Fujian | Postmenopausal women | PCR-RFLP | 77 | 57.1± 3.2 | 54 | 56.7± 2.7 | 22 | 45 | 10 |  | 20 | 30 | 4 |  | 2.57 | 0.109 |
| Wang 2012 | Hospital-  based | Shanghai | Postmenopausal women | PCR-RFLP | 1094 | 69.7± 9.3 | 2386 | 65.3± 6.4 | 350 | 624 | 120 |  | 954 | 1074 | 358 |  | 3.79 | 0.052 |
| Wu 2005 | Hospital-  based | Guangdong | Postmenopausal women | PCR-RFLP | 73 | 64.6± 8.3 | 61 | 60.7± 7.2 | 28 | 39 | 6 |  | 29 | 29 | 3 |  | 1.60 | 0.206 |
| Sui 2008 | Hospital-  based | Heilongjiang | Men and women | PCR-RFLP | 272 | 33.2± 10.4 | 208 | 33.8± 11.6 | 75 | 132 | 65 |  | 56 | 101 | 51 |  | 0.17 | 0.683 |
